# Biofilm-mediated immune dysregulation in chronic pulmonary diseases: mechanisms and clinical implications

**DOI:** 10.3389/fmicb.2025.1736384

**Published:** 2026-01-12

**Authors:** Xiao Yu, Yidan Zhang, Yiwen Yao, William C. Cho, Anquan Shang

**Affiliations:** 1 Department of Otolaryngology-Head and Neck Surgery, Shanghai Sixth People’s Hospital Affiliated to Shanghai Jiao Tong University School of Medicine, Shanghai, China; 2 Department of Respiratory and Critical Care Medicine, Shanghai Chest Hospital, Shanghai Jiao Tong University School of Medicine, Shanghai, China; 3 Department of Internal Medicine V-Pulmonology, Allergology, Respiratory Intensive Care Medicine, Saarland University Hospital, Homburg, Germany; 4 Department of Clinical Oncology, Queen Elizabeth Hospital, Kowloon, Hong Kong SAR, China; 5 Department of Laboratory Medicine, Affiliated Lianyungang Clinical College of Nantong University, Lianyungang, Jiangsu, China

**Keywords:** airway remodeling, biofilm, chronic pulmonary disease, immune dysregulation, inflammation

## Abstract

Microbial biofilms are increasingly recognized as critical modulators of chronic airway inflammation and immune dysregulation in pulmonary diseases. This review summarizes current evidence on how biofilm formation and persistence alter host immune responses, contributing to the pathogenesis of chronic lung disorders. We first outline the characteristics of respiratory biofilms and the major pathogens involved. We then discuss how biofilms reshape innate and adaptive immunity—impairing pattern recognition receptor signaling, promoting neutrophil extracellular trap (NET) formation, altering macrophage polarization, and skewing T-cell differentiation. These immune alterations sustain low-grade inflammation, tissue remodeling, and immune tolerance, driving disease progression in chronic obstructive pulmonary disease, bronchiectasis, severe asthma, and even lung cancer. We further highlight emerging diagnostic biomarkers and therapeutic approaches targeting biofilm-associated immune pathways, including combined antibiofilm and immunomodulatory strategies. Finally, we identify key knowledge gaps and propose future research directions, emphasizing multi-omics approaches and personalized interventions to better define and target biofilm-driven immune dysregulation in chronic respiratory diseases.

## 1 Introduction

Chronic pulmonary diseases represent a significant global health burden, characterized by persistent inflammation, progressive tissue damage, and recurrent infections. While the role of individual pathogens in acute exacerbations is well-established, the persistent nature of many chronic lung conditions is increasingly linked to airway microbial biofilms (Vestby et al., 2020). Biofilms are complex communities of microorganisms encased in an extracellular polymeric substance (EPS) matrix, which confer resistance to host immune defenses and antimicrobial therapies (Wu et al., 2014; Sharma et al., 2019; Luo et al., 2021). This persistent microbial presence engages in complex interplay with host immunity, leading to chronic immune dysregulation that drives the pathogenesis of diseases such as chronic obstructive pulmonary disease (COPD), bronchiectasis, severe asthma, and lung cancer (Choi E. et al., 2023; Yeung et al., 2024; Montanari et al., 2025).

The respiratory tract, with its diverse microbiota and intricate immune surveillance mechanisms, provides a unique environment for biofilm formation. Pathogens such as *Pseudomonas aeruginosa*, *Haemophilus influenzae*, and *Staphylococcus aureus* are frequently implicated in chronic respiratory infections, often adopting a biofilm lifestyle that enables long-term colonization and evasion of eradication (Short et al., 2021; Borisova et al., 2025; De Angelis et al., 2025). The transition from planktonic growth to biofilm formation alters bacterial physiology, including gene expression, metabolic activity, and virulence factor production, which in turn shapes host-pathogen interactions and immune responses (Moradali et al., 2017; Riquelme et al., 2020b).

This review systematically explores the multifaceted mechanisms by which biofilms mediate immune dysregulation and contribute to chronic inflammatory and remodeling processes in lung diseases. We first outline the fundamental aspects of biofilm formation and the key characteristics that enable their persistence in the respiratory tract, highlighting major colonizing bacterial species. We then examine how biofilms influence both innate and adaptive immunity, including the impairment of pattern recognition receptor (PRR) signaling, dysregulation of NET formation, imbalance in macrophage polarization, and skewing of T-cell subset distributions. These perturbations collectively foster chronic inflammation and immune tolerance.

The downstream consequences of such immune dysregulation, including tissue remodeling, are discussed in the context of specific chronic pulmonary conditions such as COPD, bronchiectasis, refractory asthma, and lung cancer. We also summarize emerging biomarkers and advanced methodologies for detecting biofilm-associated immune activity, which may offer diagnostic and prognostic utility. Furthermore, we evaluate recent advances in combined anti-biofilm and immunomodulatory therapeutic strategies, highlighting challenges and opportunities in this evolving field. Finally, we address current research limitations and outline future directions, emphasizing the need for multi-omics integration and personalized interventions to effectively combat biofilm-mediated immune dysregulation in chronic respiratory diseases.

## 2 Biofilm formation and characteristics in the respiratory tract

Microbial biofilms are a ubiquitous mode of bacterial growth, representing a highly organized and protected lifestyle that significantly contributes to the persistence of infections, particularly in chronic pulmonary diseases (Vestby et al., 2020; Su et al., 2022). Unlike their planktonic counterparts, biofilm-dwelling bacteria are encased within an EPS matrix, which is a complex mixture of polysaccharides, proteins, extracellular DNA (eDNA), and lipids (Flemming et al., 2022). This matrix serves multiple crucial functions, including providing structural integrity, acting as a diffusion barrier against antimicrobial agents and immune cells, facilitating nutrient acquisition, and mediating cell-to-cell communication and adhesion (Silva et al., 2019; Luo et al., 2021; Flemming et al., 2022). The formation of a biofilm is a dynamic, multi-step process, often conceptualized as a life cycle involving initial attachment, microcolony formation, maturation, and eventual dispersal of planktonic cells that can colonize new sites (Lister and Horswill, 2014; Sauer et al., 2022). However, the classic mushroom-shaped model of biofilm development has been expanded to encompass diverse, often non-surface attached, scenarios, highlighting the flexibility and adaptability of this microbial strategy (Sauer et al., 2022). For instance, an alternative “free-floating” or planktonic biofilm model begins with the aggregation of bacterial cells in the liquid phase, proceeds with the development of a structured extracellular matrix around the aggregate, and culminates in a mature, suspended community capable of dispersal, without ever attaching to a solid substratum (Sauer, 2023).

The respiratory tract, with its moist, nutrient-rich environment and diverse epithelial surfaces, provides an ideal niche for biofilm development. Key pathogenic bacteria frequently implicated in chronic pulmonary infections, such as *Pseudomonas aeruginosa*, *Haemophilus influenzae*, and *Staphylococcus aureus*, have evolved sophisticated mechanisms to form and maintain biofilms within the airways, contributing significantly to disease chronicity and exacerbations (Welp and Bomberger, 2020; Short et al., 2021; Borisova et al., 2025; De Angelis et al., 2025; Table 1).

**TABLE 1:** Major biofilm-forming pathogens in the respiratory tract.

Pathogen	Key biofilm characteristics and EPS components	Immune evasion mechanisms	Associated chronic pulmonary diseases	References
*Pseudomonas aeruginosa*	- Produces alginate, Psl, Pel exopolysaccharides - Lectin LecB stabilizes matrix - Metabolic adaptation to host itaconate enhances EPS production - Mucoid conversion, hypermutator phenotypes	- Physical barrier of EPS - Modification of LPS to reduce TLR4 recognition - Protection from phagocytosis and antibiotics	- Cystic fibrosis - Bronchiectasis - Severe asthma - COPD	Moradali et al., 2017; Borisova et al., 2025
*Haemophilus influenzae*	- Adherence to epithelial cells - Reduced metabolism within EPS - Increased ECM production	- Enhanced tolerance to antibiotics and immune clearance due to EPS and metabolic state	- COPD - Bronchiectasis	Short et al., 2021; De Angelis et al., 2025
*Staphylococcus aureus*	- Polysaccharide intercellular adhesin (PIA) via ica operon - ica-independent mechanisms - EPS synthesis promoted by host itaconate - Binds to human ECM proteins - Polymicrobial interactions	- Physical barrier of EPS - Protection from antibiotics and phagocytosis - Modulation of host immunometabolism	- Severe asthma - Cystic fibrosis - COPD	Tomlinson et al., 2021; Vila et al., 2021

The EPS matrix itself is a dynamic and complex entity, serving as a protective shield and a communication hub. It includes not only polysaccharides but also proteins, lipids, and eDNA, which collectively stabilize the matrix, store nutrients, and protect enzymes (Flemming et al., 2022). The composition and structure of the EPS can vary significantly depending on the bacterial species, environmental conditions, and even the presence of other microbes in polymicrobial biofilms (Periasamy et al., 2015). For example, *Vibrio cholerae* utilizes a biofilm matrix including mannose-sensitive hemagglutinin pili, toxin-coregulated pili, and TcpF to encase and kill immune cells, demonstrating a multicellular predation strategy (Vidakovic et al., 2023). This stands in contrast to its biofilm formation on abiotic surfaces or epithelial cells, which typically relies on a distinct set of matrix components such as the Vibrio polysaccharide (VPS) and the proteins RbmA, RbmC, and Bap1 for structural integrity and adhesion (Berk et al., 2012). The shift to a pilus-dominated matrix when targeting immune cells highlights that biofilm composition is not static but is adaptively remodeled in response to specific host surfaces and defensive pressures (Vidakovic et al., 2023). The ability of bacteria to form biofilms is also linked to respiration, a universal energy acquisition method, which can drive bacterial speciation and social microbial interactions within the biofilm niche (Martín-Rodríguez, 2023). Moreover, bacterial extracellular vesicles (BEVs) are increasingly recognized as important components of the biofilm microenvironment, playing dual roles in eliciting and suppressing biofilm formation and virulence, further adding to the complexity of biofilm biology (Jeong et al., 2024). Understanding these intricate mechanisms of biofilm formation, their structural characteristics, and the specific roles of major respiratory pathogens is foundational to comprehending how they mediate immune dysregulation and drive chronic pulmonary diseases.

## 3 Biofilm-mediated dysregulation of innate immunity

**FIGURE 1:**
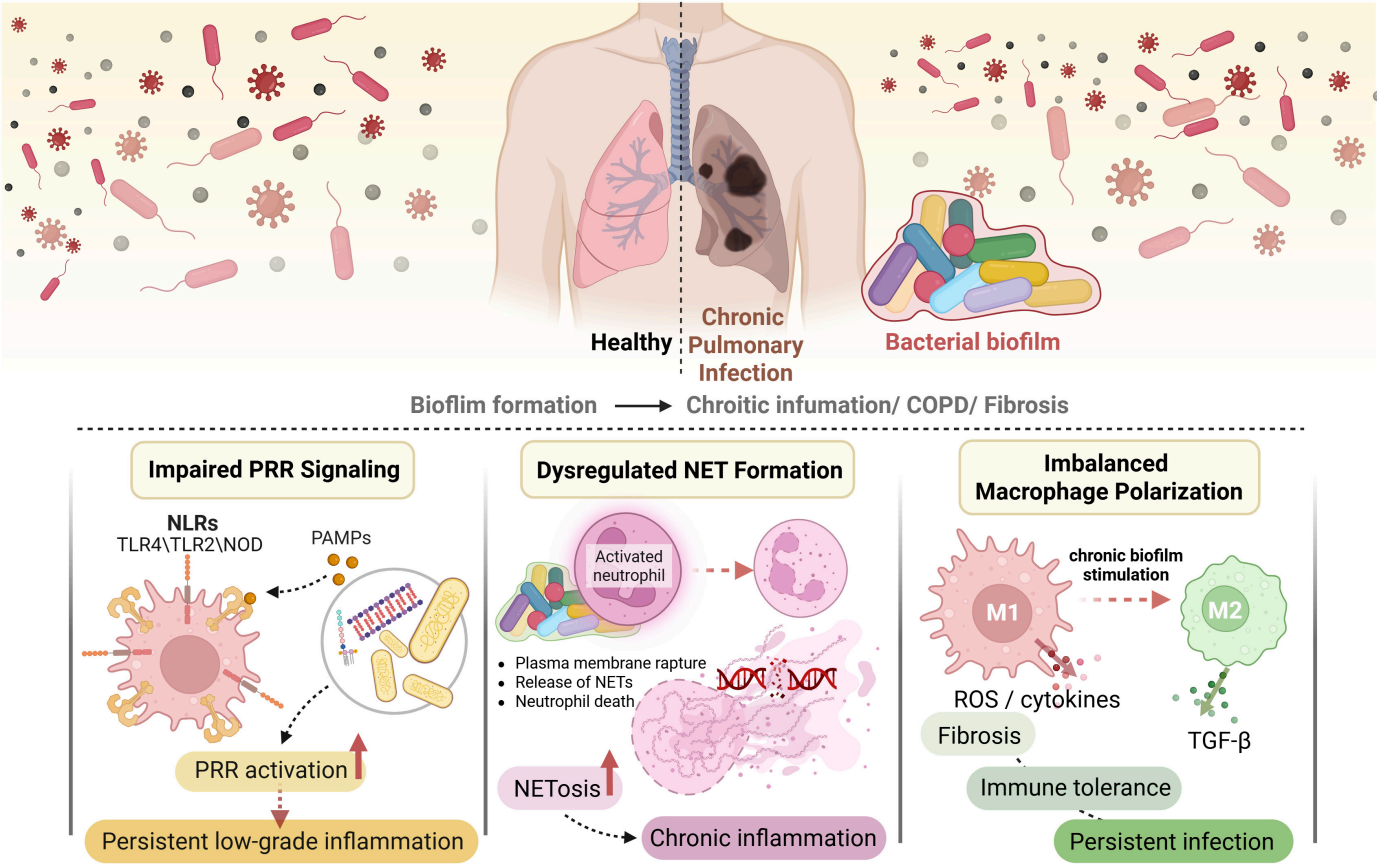
Biofilm-mediated dysregulation of innate immune responses in chronic pulmonary infection.

The innate immune system serves as the first line of defense against invading pathogens, relying on conserved PRR to detect microbial components and initiate inflammatory responses. However, in the context of chronic pulmonary infections, biofilms actively subvert and dysregulate these innate immune mechanisms, leading to persistent inflammation and impaired pathogen clearance. This dysregulation is a critical factor in the pathogenesis and progression of chronic lung diseases (Figure 1).

### 3.1 Impaired PRR signaling

Biofilms can significantly impair PRR signaling, thereby dampening effective host immune responses. PRRs, such as toll-like receptors (TLRs) and NOD-like receptors (NLRs), recognize pathogen-associated molecular patterns (PAMPs) like lipopolysaccharide (LPS), peptidoglycan, and bacterial DNA. However, the biofilm matrix acts as a physical and chemical barrier, limiting the accessibility of PAMPs to host immune cells and their PRRs (Luo et al., 2021; Flemming et al., 2022). For instance, the dense EPS matrix can sequester PAMPs, preventing their efficient recognition by immune cells, which leads to a reduced or altered inflammatory signal that is insufficient for pathogen clearance but sufficient to maintain a state of chronic low-grade inflammation (Sharma et al., 2019).

Beyond physical shielding, biofilms can actively modulate PRR signaling pathways. Some biofilm-forming pathogens, such as *Pseudomonas aeruginosa*, can alter their PAMPs or produce specific factors that interfere with PRR activation. For example, *P. aeruginosa* can modify its LPS structure, making it less immunogenic and thus less effectively recognized by TLR4, contributing to immune evasion (Riquelme et al., 2020a). Furthermore, the biofilm lifestyle itself, characterized by altered metabolic states and gene expression, can lead to the production of different PAMPs or virulence factors compared to planktonic bacteria, which may elicit a distinct and often less effective immune response (Moradali et al., 2017). The host’s response to these modified PAMPs or biofilm-specific signals can become dysregulated, leading to a state of immune tolerance or an ineffective inflammatory response that fails to resolve the infection. The activation of NLRs, such as NLRC5, which senses NAD+ depletion and drives inflammatory cell death (PANoptosis), could also be modulated by biofilm-forming bacteria, although direct evidence linking biofilm to NLRC5 dysregulation is still emerging (Sundaram et al., 2024). Similar mechanisms of regulated cell death and immune infiltration have been implicated in chronic inflammatory conditions beyond the lung, such as in heart failure, where necroptosis-related genes correlate with immune cell dysregulation (Zhu et al., 2025). The complex interplay between host-derived immune signaling metabolites, like itaconate, and bacterial metabolism further exemplifies this dysregulation, where *P. aeruginosa* can utilize host itaconate to promote biofilm formation, effectively turning a host defense mechanism into a bacterial advantage (Riquelme et al., 2020b; Tomlinson et al., 2021). This intricate modulation of PRR signaling by biofilms creates an environment where pathogens can persist, while the host immune system remains chronically activated but ineffective.

### 3.2 Dysregulated NET formation

Neutrophils are crucial innate immune cells that respond rapidly to bacterial infections. One of their potent antimicrobial strategies is the formation of NETs, which are networks of decondensed chromatin decorated with antimicrobial proteins that trap and kill bacteria (Li et al., 2023). While NETs are essential for clearing many infections, their dysregulated formation in response to biofilms can contribute to chronic inflammation and tissue damage in the lung.

Biofilms can both induce and evade NET formation. On one hand, the persistent presence of bacteria within a biofilm, along with the release of bacterial components and inflammatory mediators, can trigger excessive or prolonged NETosis (Li et al., 2023). However, the very structure of the biofilm provides protection against NETs. The EPS matrix can physically entrap NETs, preventing them from effectively reaching and killing bacteria within the biofilm (Sharma et al., 2019). Moreover, some biofilm-forming bacteria produce enzymes, such as DNases, that degrade the eDNA backbone of NETs, thereby disarming this host defense mechanism (Li et al., 2022; Wang et al., 2023). For instance, the combined action of enzymes like proteinase K, dispersin B, and DNase I has been shown to significantly reduce biofilm formation and disrupt the EPS matrix, suggesting that bacterial enzymes with similar activities could counteract host NETs (Li et al., 2022; Wen et al., 2022).

The dysregulation of NETs in chronic pulmonary diseases is a double-edged sword. While ineffective against biofilm-embedded bacteria, excessive NET formation can contribute to tissue damage and inflammation. NETs contain highly pro-inflammatory molecules, including histones and proteases, which can exacerbate lung injury and contribute to airway remodeling (Li et al., 2023). In conditions like COPD, where chronic inflammation and tissue destruction are hallmarks, dysregulated NETosis could play a significant role in perpetuating the inflammatory cycle and contributing to lung pathology (Xu et al., 2024; Lan et al., 2025). The presence of specific lncRNAs has been associated with NET formation pathways in diseases like pulmonary tuberculosis, suggesting that biofilm-induced immune responses might involve complex gene regulatory mechanisms that influence NETosis (Li et al., 2023). Therefore, understanding how biofilms manipulate NET formation is crucial for developing therapeutic strategies that can effectively clear biofilm infections without exacerbating host tissue damage.

### 3.3 Imbalanced macrophage polarization

Macrophages are highly plastic innate immune cells that play a central role in both initiating and resolving inflammation, as well as in tissue repair. They can polarize into different phenotypes, broadly classified as M1 (pro-inflammatory, microbicidal) and M2 (anti-inflammatory, tissue repair, pro-fibrotic) macrophages, depending on the microenvironmental cues (Mo et al., 2024). Biofilms significantly influence macrophage polarization, often skewing it toward phenotypes that favor bacterial persistence and chronic inflammation rather than effective clearance.

In the context of chronic pulmonary infections, biofilms can induce a state where macrophages are either unable to effectively clear pathogens (impaired M1 function) or are driven toward an M2-like phenotype that promotes immune tolerance and tissue remodeling, including fibrosis (Kim and Oliver, 2023). For example, the persistent presence of biofilm-associated PAMPs and virulence factors, coupled with the physical barrier of the EPS matrix, can lead to “frustrated phagocytosis” where macrophages attempt to engulf biofilm aggregates but fail, resulting in prolonged activation and release of inflammatory mediators without effective bacterial killing (Vestby et al., 2020). This chronic, unresolved stimulation can exhaust M1 responses and promote a shift toward M2 polarization.

Some studies suggest that biofilm components or metabolites can directly influence macrophage reprogramming. Importantly, beyond manipulating immune cell function, biofilms can actively appropriate structural components derived from the host immune response to reinforce their own architecture. A prime example is the utilization of NET-derived eDNA. While intended to ensnare pathogens, the DNA backbone of NETs can be incorporated into the biofilm EPS, enhancing its stability and resistance (Sharma and Rajpurohit, 2024). Experimental evidence shows that neutrophils can reduce eDNA content in *Streptococcus oralis* biofilms, with bacterial DNA co-localizing with neutrophil membranes, suggesting a dynamic interplay and potential appropriation of genetic material (Almaarik et al., 2025). Furthermore, histone proteins, either from NETs or other host sources, may also be co-opted. Bacterial histone-like proteins, such as HU and H-NS, are crucial for structuring bacterial DNA and regulating genes essential for biofilm formation. It is plausible that exogenous host histones could serve similar structural or regulatory roles within the biofilm matrix, turning a defensive host molecule into a component of the microbial fortress. This strategy of assimilating host-derived building blocks like eDNA and histones exemplifies a sophisticated level of host-pathogen interaction, where the inflammatory response inadvertently supplies materials for biofilm consolidation and persistence (Oberto and Rouviere-Yaniv, 2001; Belik et al., 2008; Brahma et al., 2023). Moreover, novel nanodecoys designed to combat biofilm infections have been shown to modulate immune responses by promoting M2 macrophage reprogramming, alleviating inflammation, and promoting tissue healing in a microenvironment-adaptive manner (Li Y. et al., 2024; Mo et al., 2024). This indicates that biofilms themselves might possess mechanisms to induce such a shift, or that the chronic inflammatory milieu they create favors M2 polarization. The altered properties of alveolar macrophages, including increased mediator release and defective efferocytosis (the clearance of apoptotic cells), are key features in COPD, and these changes can be linked to chronic microbial stimulation, potentially from biofilms (Kim and Oliver, 2023). The dysregulation of lncRNAs, such as lncRNA-Cox2, has been implicated in regulating macrophage cytokine production and inflammatory mediators in COPD, suggesting a complex molecular interplay in biofilm-mediated macrophage dysregulation (Kim and Oliver, 2023). Similarly, exosomal lncRNA MEG3 derived from cigarette smoke extract-treated airway epithelial cells has been shown to promote M1 macrophage polarization and pyroptosis via TREM-1 upregulation in COPD (Wang L. et al., 2024).

An imbalanced macrophage polarization, with a bias toward M2 phenotypes, can have several detrimental consequences in chronic lung diseases. M2 macrophages are associated with immune suppression, reduced bacterial clearance, and the promotion of fibrosis and tissue remodeling, which are hallmarks of conditions like COPD and idiopathic pulmonary fibrosis (Tsukui et al., 2024). This shift creates a permissive environment for biofilm persistence and contributes to the progressive structural damage observed in chronic pulmonary diseases. Therefore, understanding the precise mechanisms by which biofilms manipulate macrophage polarization is crucial for developing immunomodulatory therapies that can restore effective host defense and resolve chronic inflammation.

## 4 Biofilm-mediated dysregulation of adaptive immunity

While innate immunity provides the immediate response to infection, adaptive immunity offers specific and long-lasting protection. However, biofilms are adept at manipulating adaptive immune responses, often leading to a state of chronic immune tolerance, ineffective pathogen clearance, and perpetuation of inflammation, which are central to the progression of chronic pulmonary diseases. This dysregulation primarily manifests as skewed T-cell subset distributions and altered B-cell responses.

T lymphocytes, particularly helper T (Th) cells, play a pivotal role in orchestrating adaptive immune responses by differentiating into various subsets that produce distinct cytokine profiles and mediate different effector functions. Biofilms can significantly skew these T-cell subset distributions, leading to an immune response that is either insufficient to clear the biofilm or contributes to chronic immunopathology.

In chronic pulmonary infections associated with biofilms, there is often an imbalance in Th1/Th2/Th17 responses. For instance, chronic stimulation by biofilm-embedded bacteria can lead to a persistent Th1 and Th17 response, which, while initially aimed at pathogen clearance, can become dysregulated and contribute to chronic inflammation and tissue damage. Th1 cells, characterized by IFN-γ production, and Th17 cells, producing IL-17, are known to activate innate immune cells and promote inflammatory processes, which, when sustained, can drive conditions like emphysema in COPD. Experimental animal models and genetic studies support the notion that these adaptive immune subsets contribute significantly to COPD pathophysiology, suggesting a potential autoimmune component in some smokers with emphysema, possibly triggered or exacerbated by chronic microbial presence (Kheradmand et al., 2023).

Conversely, biofilms can also induce a shift toward Th2 responses or regulatory T cell (Treg) dominance, which can suppress effective antimicrobial immunity and promote immune tolerance. Th2 responses, characterized by cytokines like IL-4, IL-5, and IL-13, are typically associated with allergic inflammation and can contribute to airway hyperresponsiveness and mucus overproduction, as seen in severe asthma (Tuli et al., 2021). While direct evidence linking specific biofilm components to Th2 skewing is still emerging, the chronic presence of certain bacterial species or their metabolites within the biofilm could trigger or perpetuate such responses. For example, *Pseudomonas aeruginosa* exoproteins have been shown to disrupt mucosal barriers and increase IL-6 production, potentially contributing to mucosal inflammation in asthmatic patients with chronic rhinosinusitis, which could indirectly influence T-cell differentiation (Tuli et al., 2021).

Furthermore, biofilms can promote the expansion or activation of Tregs, which are crucial for maintaining immune homeostasis and preventing autoimmunity but can also suppress effective anti-biofilm immunity. By inducing immune tolerance, Tregs might allow biofilms to persist unchallenged. The complex interaction between *Mycobacterium abscessus* and *Pseudomonas aeruginosa* in coinfection models provides a compelling example. In an invertebrate *Galleria mellonella* model, coinfection suppressed host immune responses including reduced epithelial expression of IL-6 and IL-8, leading to worse survival outcomes compared to individual infections (Campo-Pérez et al., 2025). While this highlights the potential for polymicrobial interactions to enhance virulence and suppress innate immune pathways, it is important to note that insects lack the adaptive T-cell-mediated responses central to the discussion in this section. Therefore, these findings primarily underscore biofilm-mediated suppression of innate immunity and overall host defense impairment, the relevance of which to adaptive immune dysregulation in mammalian systems requires further investigation.

The mucosal immune alterations observed at the early onset of tissue destruction in COPD also involve changes in adaptive immune cells. While early stages show CD8+ T cell accumulation and IgA pathway activation, severely affected zones exhibit upregulated myeloid cells, CD4+ T cells, B cells, MHCII, and IgA pathway genes (Fays et al., 2023). These findings suggest a dynamic and complex adaptive immune response to chronic insults, which could include biofilm presence, evolving from initial attempts at containment to a more widespread, yet ultimately ineffective, inflammatory state. The dysregulation of NK cell activation and myeloid-lymphoid imbalance, with elevated neutrophils, eosinophils, and classical monocytes, also underpins COPD progression, further indicating a broad immune dysregulation that biofilms might contribute to (Qiao et al., 2025).

In summary, biofilms orchestrate a complex manipulation of adaptive immunity, leading to skewed T-cell subset distributions that either drive chronic, ineffective inflammation or promote immune tolerance, both of which contribute to the persistence of infection and the progressive tissue damage characteristic of chronic pulmonary diseases. Understanding these intricate interactions is crucial for designing immunomodulatory therapies that can restore effective adaptive immunity against biofilm infections.

## 5 Biofilm-driven chronic inflammation and tissue remodeling

**FIGURE 2:**
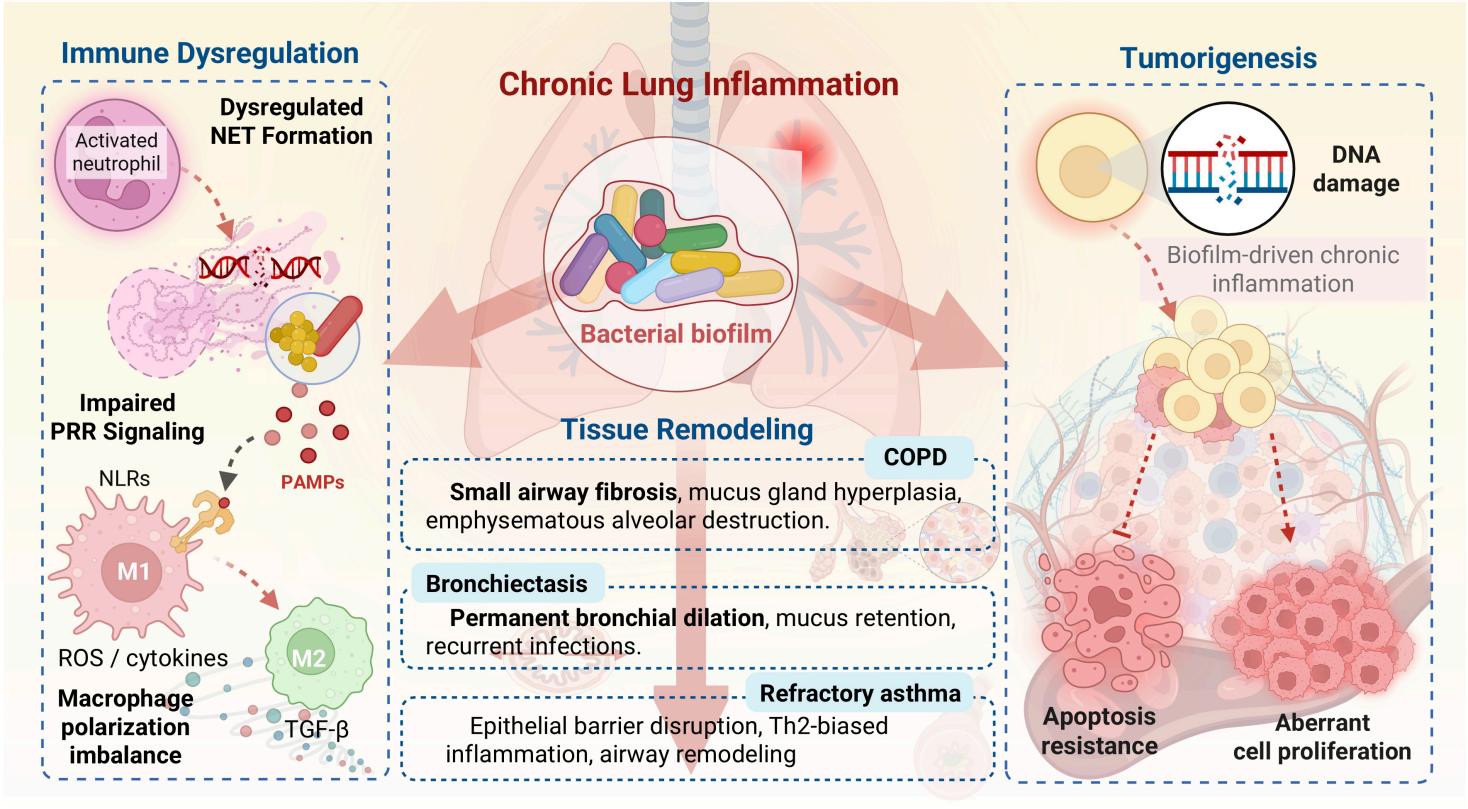
Biofilm-driven chronic inflammation and tissue remodeling in chronic pulmonary diseases.

The persistent immune dysregulation mediated by microbial biofilms in the respiratory tract is a primary driver of chronic inflammation and pathological tissue remodeling, ultimately promoting the development and progression of various chronic pulmonary diseases. This section delves into how the altered innate and adaptive immune responses, orchestrated by biofilms, translate into the clinical manifestations of conditions like COPD, bronchiectasis, refractory asthma, and even lung cancer (Figure 2).

### 5.1 COPD

COPD is a progressive lung disease characterized by persistent airflow limitation and chronic respiratory symptoms, largely driven by chronic inflammation and structural changes in the airways and lung parenchyma (Xu et al., 2024; Lan et al., 2025). Microbial biofilms, particularly those formed by *Haemophilus influenzae*, *Pseudomonas aeruginosa*, and *Staphylococcus aureus*, are increasingly recognized as significant contributors to COPD pathogenesis and exacerbations (Welp and Bomberger, 2020; Short et al., 2021; Lin et al., 2025). The chronic presence of these biofilms sustains a low-grade inflammatory state, even in stable disease, and contributes to acute exacerbations, which are critical events in disease progression.

Biofilm-mediated immune dysregulation fuels chronic inflammation in COPD through several mechanisms. The impaired PRR signaling and frustrated phagocytosis by macrophages, as discussed earlier, lead to a continuous, yet ineffective, immune activation. This results in the sustained release of pro-inflammatory cytokines and chemokines, attracting and activating various immune cells, including neutrophils, macrophages, and lymphocytes, to the airways (Fays et al., 2023; Kim and Oliver, 2023; Lan et al., 2025). Elevated neutrophils, eosinophils, and classical monocytes, along with dysregulated NK cell activation, are observed in COPD, correlating with disease severity and smoking-induced immune remodeling, which biofilms likely exacerbate (Lin et al., 2025; Qiao et al., 2025). The dysregulated NET formation, while failing to clear biofilm bacteria, releases damaging proteases and oxidants that contribute to airway and alveolar destruction, a hallmark of emphysema (Li et al., 2023).

The chronic inflammation driven by biofilms also leads to irreversible tissue remodeling. In COPD, this includes small airway fibrosis, mucus gland hyperplasia, and emphysematous destruction of alveolar walls (Fays et al., 2023; Tsukui et al., 2024). The imbalanced macrophage polarization toward M2-like phenotypes, often induced by chronic biofilm presence, can promote fibrotic processes and impair tissue repair, further contributing to structural damage (Kim and Oliver, 2023). In line with this, cigarette smoke-induced exosomal lncRNA MEG3 has been demonstrated to drive M1 polarization and pyroptosis through the SPI1/METTL3/TREM-1 axis, highlighting another pathway by which environmental exposures exacerbate macrophage dysregulation in COPD (Wang L. et al., 2024). Adaptive immune cells, particularly Th1 and Th17 subsets, activated by persistent microbial antigens, contribute to this pathology by activating innate immune cells and promoting emphysema in experimental models (Kheradmand et al., 2023). The interplay between host-microbe networks, as revealed by analyses of airway microbiota in severe COPD, shows correlations with disrupted immune-metabolic pathways, further emphasizing the systemic impact of microbial communities on disease severity (Lin et al., 2025). Moreover, immune dysregulation in COPD patients is also linked to depressive symptoms, with altered levels of BDNF, PD-1, MMP-9, and inflammatory cytokines, suggesting a broader systemic impact of chronic inflammation that could be influenced by biofilm presence (Liu et al., 2025).

### 5.2 Bronchiectasis

Bronchiectasis is a chronic respiratory condition characterized by permanent dilation of the bronchi and bronchioles, leading to impaired mucociliary clearance, recurrent infections, and a vicious cycle of inflammation and airway damage (De Angelis et al., 2025). Biofilm formation by pathogens like *Pseudomonas aeruginosa* and *Haemophilus influenzae* is a central feature in the pathophysiology of bronchiectasis, driving persistent infection and chronic inflammation (Borisova et al., 2025; De Angelis et al., 2025).

The presence of biofilms in bronchiectatic airways creates a persistent reservoir of bacteria that are highly resistant to antibiotics and host defenses. This leads to chronic bacterial colonization, which continuously stimulates the host immune system. The resulting inflammation, characterized by neutrophil influx and release of proteases, contributes to the progressive destruction of the bronchial walls, leading to further airway dilation and impaired mucus clearance, thus perpetuating the cycle (De Angelis et al., 2025). *P. aeruginosa* biofilms, with their robust EPS matrix and ability to evade immune responses, are particularly problematic, often leading to a decline in lung function and increased exacerbation frequency (Borisova et al., 2025). While H. influenzae is also a common pathogen, its role in bronchiectasis-related morbidity and progression, especially in the context of biofilm formation, requires further investigation. It is important to note that bronchiectasis can also be caused by other pathogens, including non-tuberculous mycobacteria (NTM) such as Mycobacterium avium complex. In cases of NTM infection, which account for a significant proportion of bronchiectasis cases, long-term macrolide monotherapy is strongly discouraged due to the high risk of inducing antibiotic resistance (Zhou et al., 2022). Therefore, for chronic biofilm-associated infections in bronchiectasis, including but not limited to H. influenzae, carefully considered multi-drug regimens are often a more effective and safer therapeutic approach than monotherapy to mitigate resistance risks (De Angelis et al., 2025). The interaction between different pathogens, such as the negative interaction between *P. aeruginosa* and H. influenzae, can influence microbiome dynamics and disease outcomes, highlighting the importance of understanding polymicrobial biofilms in bronchiectasis (Campo-Pérez et al., 2025; De Angelis et al., 2025).

### 5.3 Refractory asthma

While asthma is primarily an allergic disease, a subset of patients experiences severe or refractory asthma, often characterized by persistent airway inflammation, corticosteroid resistance, and frequent exacerbations, where chronic bacterial or fungal colonization, often in biofilm form, is increasingly implicated (Welp and Bomberger, 2020). Biofilms can contribute to refractory asthma by sustaining chronic inflammation, altering airway epithelial barrier function, and modulating immune responses in a way that promotes allergic sensitization or exacerbates existing inflammation.

*Pseudomonas aeruginosa* exoproteins, for example, have been shown to disrupt mucosal barriers and increase IL-6 production in asthmatic patients with chronic rhinosinusitis, potentially contributing to mucosal inflammation and exacerbating asthma symptoms (Tuli et al., 2021). The chronic presence of biofilm-associated bacteria can also trigger or perpetuate a Th2-biased immune response, leading to increased eosinophilic inflammation, mucus hypersecretion, and airway hyperresponsiveness, which are characteristic features of asthma. Moreover, the chronic immune activation by biofilms can lead to airway remodeling, including subepithelial fibrosis and smooth muscle hypertrophy, contributing to fixed airflow obstruction and reduced responsiveness to conventional therapies in severe asthma. The complex interplay of microbial communities in the respiratory tract, including common pathogens like *Staphylococcus aureus* and Moraxella catarrhalis, can become dysbiotic in chronic respiratory diseases, correlating with worsening morbidity and potentially contributing to asthma severity (Welp and Bomberger, 2020).

### 5.4 Lung cancer

Emerging evidence suggests a complex interplay between microbial biofilms, chronic inflammation, and the development and progression of various cancers, including lung cancer (Choi E. et al., 2023; Wang Y. et al., 2024; Yeung et al., 2024; Montanari et al., 2025). Biofilms can contribute to carcinogenesis through several mechanisms, including chronic inflammation, production of genotoxic metabolites, and modulation of the tumor microenvironment (TME).

Chronic inflammation, driven by persistent biofilm infections, is a well-established risk factor for cancer. The sustained release of reactive oxygen species (ROS), reactive nitrogen species (RNS), and pro-inflammatory cytokines by immune cells attempting to clear biofilms can lead to DNA damage, promote cell proliferation, and inhibit apoptosis, thereby creating a pro-carcinogenic environment (Huang et al., 2024; Ma M. et al., 2024). Biofilms also share physical and chemical traits with cancer cells, such as protection against immune responses, which can further complicate the immune landscape within the TME (Choi E. et al., 2023).

Recent studies highlight the direct role of bacteria, often in biofilm-like structures, within the tumor microenvironment. For example, *P. aeruginosa* biofilms have been shown to influence tumor survival and ferroptosis resistance in lung tumor spheroid coculture models, with bacterial siderophores like pyoverdine enhancing cancer hallmarks. This cross-kingdom cooperation suggests that targeting biofilms could be a strategy for combating cancer (Yeung et al., 2024). The tumor-associated microbiota, including fungi, often exhibits biofilm-like structures that protect against immune destruction and influence oncogene expression and progression (Choi E. et al., 2023; Wang Y. et al., 2024; Montanari et al., 2025). Understanding these interactions may lead to innovative therapeutic approaches, including using bacteria as delivery vectors, enhancing immune function, and targeting shared biofilm-cancer traits (Choi V. et al., 2023). High-resolution spatial atlases of lung cancer and chronic lung diseases are beginning to map the architectural organization of immune cell types and their microenvironmental interactions, which will be crucial for understanding the role of biofilms in this context and developing targeted therapies (Nandigama et al., 2024).

In essence, the chronic inflammatory state and pathological tissue remodeling induced by biofilm-mediated immune dysregulation are central to the progression of chronic pulmonary diseases. By sustaining inflammation, impairing tissue repair, and even contributing to oncogenic processes, biofilms represent a critical, yet often overlooked, factor in the complex etiology of these debilitating conditions.

## 6 Emerging biomarkers and methodologies for detecting biofilm-associated immune activity

Accurate detection and assessment of biofilm-associated immune activity are crucial for both diagnosing chronic pulmonary diseases and guiding therapeutic interventions. Traditional microbiological methods often fail to capture the biofilm lifestyle, leading to underestimation of their prevalence and impact. Consequently, there is a growing need for novel biomarkers and advanced methodologies that can specifically identify biofilm presence and characterize the host immune response to them.

### 6.1 Biomarkers of biofilm presence and activity

Emerging biomarkers aim to detect either components of the biofilm matrix or specific host responses indicative of chronic biofilm infection. These biomarkers can broadly be categorized into three interrelated groups based on their biological origin and diagnostic potential.

(1) Biofilm matrix components: The EPS matrix is a unique signature of biofilms. Components like specific exopolysaccharides (e.g., alginate, Psl, Pel from *P. aeruginosa*), eDNA, and biofilm-specific proteins can serve as biomarkers (Flemming et al., 2022; Gheorghita et al., 2023). Methodologies for characterizing EPS composition are evolving beyond traditional colorimetric methods, which often suffer from biases, toward more advanced analytical techniques to identify specific EPS components. Detecting these components in respiratory samples (e.g., sputum, bronchoalveolar lavage fluid) could directly indicate biofilm presence.

(2) Bacterial quorum sensing (QS) molecules: Biofilm formation and virulence are often regulated by quorum sensing (QS) systems, which involve the production and detection of small signaling molecules (Moradali et al., 2017; Sharma et al., 2019; Venkateswaran et al., 2023; Juszczuk-Kubiak, 2024). Detecting these QS molecules [e.g., autoinducers, diffusible signal factors (DSF)] in airway secretions could serve as a biomarker for active biofilm formation and communication (Deng et al., 2014). For example, N-salicyl-AAn-picolamide peptides have been shown to inhibit *P. aeruginosa* quorum sensing, highlighting the potential for QS molecules as targets for both detection and intervention (Panda et al., 2023).

(3) Host immune response signatures: Biofilm-mediated immune dysregulation leaves distinct immunological footprints. Biomarkers could include specific cytokine profiles (e.g., persistent low-grade pro-inflammatory cytokines, altered anti-inflammatory mediators), altered immune cell populations (e.g., skewed T-cell subsets, imbalanced macrophage polarization), or markers of tissue damage and remodeling (Fays et al., 2023; Kim and Oliver, 2023; Long et al., 2023; Lin et al., 2025; Qiao et al., 2025). For instance, high-dimensional immune profiling has revealed myeloid-lymphoid imbalance and NK cell hyperactivity in COPD progression, which could be linked to chronic microbial stimulation (Qiao et al., 2025). Similarly, the association of specific lncRNAs with NET formation pathways in pulmonary tuberculosis suggests their potential as novel biomarkers for infection and therapy monitoring (Li et al., 2023). Multi-modal transcriptomic analysis in COPD has also identified key genes and metabolic dysregulation, offering novel biomarkers for early intervention (Luo et al., 2024). The detection of damage-associated molecular patterns (DAMPs) and their sensing receptors, which mediate sterile inflammation, could also serve as indicators of biofilm-induced tissue damage and inflammation (Huang et al., 2024; Ma M. et al., 2024).

### 6.2 Advanced methodologies for detection and assessment

Traditional culture-based methods often underestimate biofilm presence due to the difficulty of culturing biofilm-embedded bacteria and their altered metabolic states. Advanced methodologies are therefore essential:

(1) Molecular diagnostics: Techniques like quantitative PCR (qPCR) and next-generation sequencing (NGS), including clinical metagenomics (mNGS), allow for the detection and identification of microbial species, including those that are difficult to culture, and can provide insights into microbial diversity and community structure within the airways (Chiu and Miller, 2019; Lin et al., 2025). It is important to note, however, that the accuracy and interpretability of mNGS results are highly dependent on methodological rigor. The choice of sample type is critical; for instance, bronchoalveolar lavage fluid (BALF), while clinically accessible, often has low microbial biomass, which can affect sensitivity and increase the relative impact of contaminating DNA. Studies have shown that lung tissue samples may provide a more accurate representation of the true airway microbiota compared to BALF (Baker et al., 2021). Furthermore, stringent experimental controls, including negative controls to account for reagent and environmental contamination, are essential to avoid false-positive results and bias in community profiling (Li R. et al., 2024). While mNGS offers a comprehensive view of the microbiome, its application in distinguishing planktonic from biofilm-associated bacteria or directly quantifying biofilm burden remains a challenge. However, by identifying increased pathogenic bacteria and dysregulated inflammatory signaling, mNGS can indirectly point toward biofilm-associated pathology (Lin et al., 2025).

(2) Imaging techniques: Confocal laser scanning microscopy (CLSM) and other advanced imaging modalities can directly visualize biofilms in clinical samples or *in situ* in animal models, providing information on biofilm architecture, thickness, cellular viability, and dynamic development. A significant advancement is the use of Light Sheet Fluorescence Microscopy (LSFM), which enables label-free, nondestructive, long-term imaging of living biofilms with high spatial and temporal resolution, allowing precise quantification of thickness and growth dynamics (Koch et al., 2020). Overcoming the traditional limitations of CLSM such as phototoxicity, photobleaching, and limited depth resolution in dense 3D structures new methodologies are emerging. These include advanced modalities like iCBiofilm and the integration of tissue clearing methods with techniques such as hybridization chain reaction, which enable high-resolution, precise *in situ* imaging within complex tissues and airway-derived samples (Zhang et al., 2020). For instance, microfluidic 3D lung tumor spheroid-*P. aeruginosa* models allow for the visualization of bacterial biofilms and their interaction with tumor cells (Yeung et al., 2024). *In vivo* imaging techniques, such as intravital imaging, can track biofilm dispersal and the efficacy of antibiofilm agents in real-time (Tian et al., 2020).

(3) Vibrational spectroscopy: Raman spectroscopy and infrared (IR) spectroscopy are emerging as powerful, non-invasive tools for identifying and characterizing microbes at a single-cell level, detecting antimicrobial resistance, and potentially distinguishing biofilm-associated bacteria from planktonic forms based on their distinct biochemical signatures (Rebrosova et al., 2022; Pirutin et al., 2023). These methods offer rapid, culture-independent identification and characterization, holding promise for future point-of-care diagnostics in clinical settings (Rebrosova et al., 2022).

(4) Biofilm-specific *in vitro* and *ex vivo* models: Developing sophisticated *in vitro* and *ex vivo* models that accurately mimic the complex environment of chronic pulmonary infections is crucial for studying biofilm formation and testing therapeutic interventions. Examples include murine subcutaneous catheter models for polymicrobial infections, porcine *ex vivo* skin wound models for polymicrobial biofilms, and organotypic models of peri-implant oral mucosa that differentiate commensal and pathogenic biofilm effects (Ingendoh-Tsakmakidis et al., 2019; Pati et al., 2021; Thaarup and Bjarnsholt, 2021; Vila et al., 2021). These models allow for controlled studies of biofilm dynamics, host-pathogen interactions, and the efficacy of novel antibiofilm agents, providing a platform for biomarker discovery and validation.

(5) Multi-omics approaches: The integration of genomics, transcriptomics, proteomics, and metabolomics (multi-omics) offers a comprehensive understanding of both the microbial community and the host response in chronic pulmonary diseases (Luo et al., 2024; Lin et al., 2025). By analyzing the expression of bacterial biofilm-related genes (e.g., quorum sensing genes, EPS synthesis genes) alongside host immune and inflammatory pathways, multi-omics can identify specific signatures of biofilm-mediated immune dysregulation. For example, RNA-seq analysis has revealed how *C. albicans* activates *S. aureus* biofilm formation via gene modulation, highlighting the power of transcriptomics in understanding polymicrobial interactions (Vila et al., 2021). Such integrated approaches are essential for identifying novel diagnostic biomarkers and therapeutic targets, moving toward personalized medicine in chronic lung diseases.

The development of these biomarkers and methodologies is critical for overcoming the limitations of traditional approaches and gaining a more accurate understanding of the role of biofilms in chronic pulmonary diseases. By providing precise tools for detection and characterization, they pave the way for more targeted and effective therapeutic strategies (Table 2).

**TABLE 2:** Advanced methodologies for the detection and assessment of biofilm.

Method	Core idea	Advantages	Disadvantages	References
Molecular diagnostics (qPCR, NGS, mNGS)	Detects and identifies microbial species and community structure via genetic material.	- Comprehensive view of microbiome - Identifies unculturable microbes - Insights into diversity and dysbiosis	- Difficulty distinguishing planktonic from biofilm-associated bacteria - Challenges in quantifying biofilm burden directly.	Chiu and Miller, 2019; Luo et al., 2024; Lin et al., 2025
Imaging techniques (CLSM, intravital imaging)	Directly visualizes biofilms in samples or *in situ*.	-Provides information on biofilm architecture, thickness, cellular viability, and real-time dynamics	- Requires specialized equipment - Can be invasive for *in vivo* applications - Limited to accessible sites.	Tian et al., 2020; Li et al., 2022; Yeung et al., 2024
Vibrational spectroscopy (Raman, IR)	Identifies and characterizes microbes at single-cell level based on distinct biochemical signatures.	- Rapid, non-invasive, culture-independent identification - Potential for distinguishing biofilm from planktonic forms - Point-of-care diagnostic promise.	- Requires sophisticated spectral analysis - Specificity can be challenging in complex samples.	Rebrosova et al., 2022; Pirutin et al., 2023
Multi-omics approaches (genomics, transcriptomics, proteomics, and metabolomics)	Integrates data from microbial and host perspectives to understand complex interactions.	- Comprehensive understanding of microbial community and host response - Identifies novel biomarkers and therapeutic targets - Reveals biofilm-related gene expression and host immune pathways.	- High data complexity - Requires advanced bioinformatics for integration and interpretation.	Vila et al., 2021; Luo et al., 2024; Lin et al., 2025

## 7 Combined anti-biofilm and immunomodulatory therapies

Given the inherent resistance of biofilms to conventional antibiotics and the complex immune dysregulation they induce, effective treatment of biofilm-associated chronic pulmonary diseases necessitates innovative therapeutic strategies. A promising approach involves combining anti-biofilm agents with immunomodulatory therapies to simultaneously disrupt the biofilm, enhance antibiotic efficacy, and restore effective host immunity.

### 7.1 Anti-biofilm strategies

Anti-biofilm strategies primarily aim to prevent biofilm formation, disrupt mature biofilms, or enhance the penetration and efficacy of antibiotics within the biofilm matrix. These approaches encompass a diverse range of molecular, biochemical, and nanotechnological interventions designed to target different stages and components of biofilm development (Table 3).

**TABLE 3:** Overview of emerging anti-biofilm strategies and their mechanistic targets.

Strategy	Core mechanism	Advantages	Challenges and considerations	References
Biofilm dispersal enzymes	Degrade specific EPS components (polysaccharides, proteins, eDNA) to destabilize biofilm structure.	- Disrupts mature biofilms - Releases embedded bacteria - Potentiates antibiotics and host immune clearance	- Specificity to EPS components - Stability and delivery *in vivo* - Potential for off-target effects	Baker et al., 2016; Li et al., 2022; Wang et al., 2023
Antimicrobial peptides (AMPs)	- Target bacterial membranes - Inhibit biofilm formation - Disrupt mature biofilms - Enhance antibiotic efficacy.	- Broad-spectrum activity - Potent against MDR organisms - Synergistic with conventional antibiotics	- Stability *in vivo* - Cytotoxicity - Delivery optimization - Cost of synthesis	Mahlapuu et al., 2016; Masihzadeh et al., 2023; Ridyard et al., 2023; Roque-Borda et al., 2025
Quorum sensing modulators	Interfere with bacterial cell-to-cell communication, regulating biofilm formation and virulence.	- Prevents biofilm maturation - Renders bacteria more susceptible to antibiotics and immune clearance - Less prone to resistance development	- Specificity to QS systems - Efficacy against established biofilms - Delivery to infection site	Deng et al., 2014; Sharma et al., 2019; Juszczuk-Kubiak, 2024; Shariati et al., 2024
Nanotherapeutic strategies	Nanoparticles enhance drug delivery, penetrate biofilm matrix, provide intrinsic antibiofilm properties.	- Targeted delivery - Improved penetration - Reduced systemic toxicity - Multifunctional theranostic platforms	- NP safety and biocompatibility - Optimization of penetration - Targeted delivery to specific biofilm sites	Blanco-Cabra et al., 2024; Li Y. et al., 2024; Wang L. et al., 2024; Zou et al., 2025
Novel antibiotics and combinatorial approaches	- New antibiotic classes targeting biofilm-essential mechanisms - Synergistic use of anti-biofilm agents with conventional antibiotics	- Potent activity against resistant strains - Overcomes biofilm-mediated resistance - Enhanced efficacy	- Development of new resistance - Toxicity of combinations - Pharmacokinetics in biofilm context	Su et al., 2020; Behera et al., 2023; Sharafi et al., 2024; Zampaloni et al., 2024

(1) Biofilm dispersal enzymes: Enzymes that degrade specific components of the EPS matrix are highly effective in disrupting biofilms. Glycoside hydrolases, proteases, and deoxyribonucleases (DNases) can break down polysaccharides, proteins, and eDNA, respectively, thereby destabilizing the biofilm structure and releasing embedded bacteria, making them more susceptible to antibiotics and host immune cells (Baker et al., 2016; Li et al., 2022; Wang et al., 2023). For instance, *P. aeruginosa* exopolysaccharide biosynthetic glycoside hydrolases like PelAh and PslGh can selectively degrade the biofilm matrix, inhibiting formation and disrupting pre-existing biofilms, while also potentiating antibiotics and enhancing neutrophil killing (Baker et al., 2016). Similarly, combined lipase, cellulase, and proteinase K enzymes have shown synergistic effects in inhibiting and eradicating *Vibrio parahaemolyticus* biofilms by disrupting the EPS matrix and suppressing gene expression (Li et al., 2022). However, the effective delivery of these macromolecular enzymes to the protected biofilm niche remains a key translational challenge. Innovative delivery systems are being developed to protect enzymatic activity from denaturation and rapid clearance, and to ensure site-specific action. Strategies include the encapsulation of enzymes within nanoparticles or liposomes, their incorporation into hydrogels for localized release, and the engineering of bacteriophages for targeted delivery (Lu et al., 2023; Dsouza et al., 2024; Wang X. et al., 2024). These carriers can enhance enzyme stability, provide controlled release kinetics, and in some cases, be functionalized with targeting moieties to improve accumulation at the biofilm site (Afrasiabi and Partoazar, 2024). The use of such enzymes, either alone or in combination, represents a powerful strategy to overcome biofilm resistance. A critical consideration is that dispersion may create a distinct, transient cellular state with unpredictable antibiotic susceptibility, and uncontrolled dispersion could theoretically increase the risk of bacteremia (Su et al., 2022). Key translational challenges for enzyme therapies include susceptibility to denaturation and rapid clearance *in vivo*, potential immunogenicity, and the need for targeted delivery systems to protect enzymatic activity and ensure site-specific action (Thorn et al., 2021).

(2) Antimicrobial peptides (AMPs): AMPs are a diverse class of naturally occurring peptides with broad-spectrum antimicrobial activity, often targeting bacterial membranes, and many also exhibit potent anti-biofilm properties (Mahlapuu et al., 2016; Yasir et al., 2018; Galdiero et al., 2019; Roque-Borda et al., 2025). AMPs can inhibit biofilm formation, disrupt mature biofilms, and enhance the efficacy of conventional antibiotics, even against multidrug-resistant (MDR) organisms (Ridyard and Overhage, 2021; Neto et al., 2022; Pontes et al., 2022; Bellavita et al., 2023; Masihzadeh et al., 2023; Roque-Borda et al., 2025). For example, the natural compound prodigiosin has demonstrated strong antibiofilm activity against *Pseudomonas aeruginosa*, a key pathogen in chronic lung infections, by inhibiting biofilm formation and disrupting mature biofilms. Importantly, in a murine model of chronic pulmonary infection, PG also modulated host immune responses, such as reducing IL-1β, IL-6, TNF-α pro-inflammatory cytokines, highlighting its dual role in targeting both the biofilm and the associated dysregulated inflammation (Ma Z. et al., 2024). Human peptide LL-37 also exhibits broad-spectrum antibacterial and anti-biofilm activities, and its efficacy can be optimized through structural modifications or synergistic combinations with other agents (Ridyard and Overhage, 2021; Ridyard et al., 2023). Peptoids, which are peptidomimetics, also show promising antibacterial and antibiofilm activity, with their self-assembly properties impacting their biological effects (Nielsen et al., 2022). Melittin, a bee venom peptide, has shown synergistic antibacterial and antibiofilm effects with oxacillin against MRSA, suggesting its potential as a new antibacterial agent (Pereira et al., 2023). Challenges remain in optimizing AMP stability, reducing cytotoxicity, and improving delivery, but ongoing research is addressing these limitations (Mahlapuu et al., 2016; Roque-Borda et al., 2025). While extensively studied *in vitro* and in animal models, their clinical translation faces hurdles including limited effectiveness in complex *in vivo* milieus, potential cytotoxicity to host cells at therapeutic doses, susceptibility to proteolytic degradation, and high production costs (Abdelhamid and Yousef, 2023).

(3) Quorum sensing modulators (QSMS): QSMs interfere with bacterial cell-to-cell communication, which is crucial for biofilm formation, virulence factor production, and antibiotic resistance (Sharma et al., 2019; Lazar et al., 2021; Venkateswaran et al., 2023; Juszczuk-Kubiak, 2024). By blocking QS, these agents can prevent biofilm maturation and render bacteria more susceptible to antibiotics and immune clearance (Shariati et al., 2024). Natural compounds like curcumin, carvacrol, and thymol have shown inhibitory effects on *P. aeruginosa* quorum sensing, offering promising avenues for non-antibiotic strategies (Shariati et al., 2024). The DSF quorum sensing signal and related molecules have also been shown to enhance antibiotic efficacy against various bacterial pathogens, possibly by reducing resistance and biofilm formation (Deng et al., 2014). Their development is challenged by the need to achieve and maintain effective concentrations at the infection site, potential metabolic instability, and the complexity of redundant bacterial signaling systems which may limit efficacy and potentially drive resistance (Naga et al., 2023).

(4) Nanotherapeutic strategies: Nanoparticles (NPs) offer a versatile platform for combating biofilms by enhancing drug delivery, penetrating the biofilm matrix, and providing intrinsic antibiofilm properties (Lv et al., 2022; Blanco-Cabra et al., 2024; Dsouza et al., 2024; Wang X. et al., 2024; Zou et al., 2025). Strategies include designing NPs that target biofilm components, carry EPS-degrading enzymes, or deliver antimicrobial agents directly into the biofilm (Lv et al., 2022; Wang X. et al., 2024). For example, zwitterionic micelles can self-target and disperse infectious biofilms, enhancing antibiotic efficacy *in vitro* and *in vivo* (Tian et al., 2020). Metallo-polymeric nanodecoys (MPNs) have been developed to eradicate bacterial biofilms and modulate immune responses, offering an efficient alternative to antibiotics (Li Y. et al., 2024). Supramolecular assemblies of mesoporous silica nanoparticles can co-deliver antimicrobial peptides and antibiotics for synergistic eradication of pathogenic biofilms (Yu et al., 2020). Multifunctional theranostic nanoplatforms, such as MnO2-BSA/PEG-Ce6 NSs, have been designed for dual-mode imaging and hypoxia-relief-enhanced photodynamic therapy of bacterial biofilm infections, demonstrating enhanced efficacy in mouse models (Xiu et al., 2020). Gold nanoparticles tethering antimicrobial peptides have also shown potent broad-spectrum antimicrobial and antibiofilm activities (Rajchakit et al., 2024). Nanozymes, with their catalytic activity, are also being explored for remodeling the immune microenvironment and disrupting biofilms, offering potential for precise immune regulation (He and Zhang, 2025). Challenges include ensuring NP safety, optimizing penetration, and achieving targeted delivery (Afrasiabi and Partoazar, 2024; Blanco-Cabra et al., 2024). Although a rapidly advancing field with compelling preclinical proof-of-concept, nanomedicine for pulmonary biofilms faces significant translational barriers. These include ensuring long-term safety and biocompatibility, precise control over biodegradation, potential for unintended pulmonary inflammation, scalability of manufacturing, and navigating regulatory pathways for complex nanocarriers. A major limitation is that existing materials and delivery strategies often struggle with poor biocompatibility, insufficient local drug concentrations at the biofilm site, and unspecific interactions in the biological environment (Choi V. et al., 2023).

(5) Novel antibiotics and combinatorial approaches: New antibiotic classes that specifically target bacterial mechanisms essential for biofilm formation or survival are being developed. For example, a novel antibiotic class targeting the lipopolysaccharide transporter (LptB2FGC complex) has shown potent activity against carbapenem-resistant Acinetobacter baumannii, representing a promising treatment for invasive infections that often involve biofilms (Zampaloni et al., 2024). In the context of carbapenem-resistant Klebsiella pneumoniae infections, a recent propensity score-matched multicenter study demonstrated that ceftazidime/avibactam-based regimens were associated with higher clinical efficacy and microbiological clearance, along with a lower incidence of acute kidney injury, compared to polymyxin B-based regimens, highlighting its potential as a preferred therapeutic option against difficult-to-treat biofilm-associated infections (Zhuang H.-H. et al., 2024). Combinatorial therapy, where anti-biofilm agents are used in conjunction with conventional antibiotics, is a highly effective strategy, as demonstrated by the synergistic effects of various AMPs with antibiotics like ciprofloxacin, oxacillin, and gentamicin against *Staphylococcus aureus* and other pathogens (Häring et al., 2022; Neto et al., 2022; Behera et al., 2023; Pereira et al., 2023; Sharafi et al., 2024). Dissolvable microneedles coupled with nanofiber dressings have also been developed to effectively deliver antimicrobial peptides and antibiotics, eradicating biofilms in chronic wounds (Su et al., 2020). Translating these advanced delivery platforms for pulmonary biofilm infections represents a promising yet challenging frontier. The core principles of localized, sustained drug release and enhanced penetration are highly relevant to lung therapy. Research is actively exploring the adaptation of these technologies for respiratory use. This includes developing inhalable nanofiber formulations or biocompatible microneedle-like patches for transthoracic delivery, aimed at achieving high local drug concentrations in the lung while minimizing systemic exposure. Key considerations for pulmonary adaptation involve engineering particle size and aerodynamics for effective airway deposition, ensuring material biodegradability and safety in the delicate alveolar environment, and designing systems responsive to the unique pathophysiology of diseases like COPD or bronchiectasis (Su et al., 2020; Blanco-Cabra et al., 2024; Wang L. et al., 2024). While direct clinical application to lung biofilms requires further development, the success in wound models provides a strong foundational proof-of-concept for innovative local drug delivery strategies against entrenched infections. The clinical implications of these combined therapies are substantial, offering hope for improved outcomes in patients with chronic pulmonary diseases. Challenges include avoiding the rapid emergence of resistance, managing potential increased toxicity from drug combinations, and demonstrating superior efficacy over standard care in rigorous clinical trials (Abdelhamid and Yousef, 2023).

### 7.2 Immunomodulatory therapies

Beyond directly targeting biofilms, immunomodulatory therapies aim to correct the biofilm-mediated immune dysregulation, restoring effective host defense mechanisms and reducing chronic inflammation.

(1) Targeting inflammation: Strategies to reduce chronic inflammation include anti-inflammatory drugs, but more targeted approaches are needed for biofilm-associated inflammation. For instance, bimetal-phenolic frameworks have been shown to modulate immune responses and reduce inflammation, in addition to their direct antibacterial and biofilm dispersal effects (Wang et al., 2025). Similarly, sulfur vacancy-rich Bi2S3-X@PDA heterojunctions with light-controlled ROS generation and elimination can combat biofilm infection and inflammation by downregulating pro-inflammatory factors and promoting M2 macrophage reprogramming (Mo et al., 2024). Peptoids have also demonstrated anti-inflammatory effects comparable to conventional antibiotics, alongside their antimicrobial and antibiofilm activities (Cafaro et al., 2023). Another compelling immunomodulatory strategy involves the use of antibodies targeting the DNABII family of bacterial DNA-binding proteins, critical structural components of the extracellular DNA scaffold in biofilms (Novotny et al., 2020). These antibodies rapidly destabilize the biofilm matrix, releasing resident bacteria into a transient but highly vulnerable state (Jurcisek et al., 2022). Crucially, bacteria newly released in this manner are not only rendered more susceptible to conventional antibiotics but are also preferentially cleared by the host’s innate immune effectors (Wilbanks et al., 2023). For instance, anti-DNABII treatment can enhance neutrophil-mediated killing by boosting phagocytosis, NETosis, and chemotaxis, in part through elevated IL-8 secretion (Zhuang L. et al., 2024). This dual mechanism direct biofilm disruption coupled with the sensitization of bacteria to host defenses has demonstrated therapeutic efficacy in resolving recalcitrant infections, including in preclinical models of pulmonary disease. Their long-term safety profile, especially concerning precise immunomodulation without causing immunosuppression, requires thorough investigation (Jurcisek et al., 2025). Repurposing existing immunomodulators (e.g., macrolides) for biofilm diseases has more clinical evidence, albeit with known side-effect profiles (Ryu et al., 2023).

(2) Enhancing host immunity: This involves boosting specific arms of the immune system that are suppressed or ineffective against biofilms. For example, strategies to activate the immune system against fungal biofilms are being explored, which could be extended to bacterial biofilms (Mannan et al., 2024). Nanoparticle-based strategies that modulate the immune microenvironment, such as inducing immunogenic cell death, activating innate immune pathways, or reprogramming macrophage polarization, are emerging as promising avenues (Li Y. et al., 2024; Mo et al., 2024; He and Zhang, 2025). Thiolated chitosan nanoparticles encapsulating nisin and selenium have shown immunomodulatory effects by decreasing pro-inflammatory cytokines and increasing anti-inflammatory cytokines, while reducing TLR2 and TLR4 expression (Derakhshan-Sefidi et al., 2024). Major limitations include the risk of exacerbating inflammation or autoimmunity, the highly context-dependent and heterogeneous nature of immune responses in chronic disease, and the difficulty in achieving precise, localized immunomodulation (Cappelli and Shah, 2020).

(3) Modulating immune dysregulation: Addressing specific aspects of immune dysregulation, such as skewed T-cell responses or defective macrophage function, could improve outcomes. For instance, understanding the role of lncRNA-Cox2 in macrophage cytokine regulation in COPD could lead to targeted interventions to restore effective innate immune responses (Kim and Oliver, 2023). Similarly, modulating the PD-1/MMP-9 axis has been suggested as a therapeutic strategy for depressive symptoms in AECOPD patients, which are linked to neuroinflammation and immune dysregulation (Liu et al., 2025). The primary challenge lies in developing safe and effective modulators that can precisely correct immune defects without disrupting essential immune homeostasis, which will require extensive target validation and safety profiling (Garduno and Martín-Loeches, 2024).

### 7.3 Clinical implications and challenges

The clinical implications of these combined therapies are substantial, offering hope for improved outcomes in patients with chronic pulmonary diseases. By simultaneously attacking the biofilm and correcting immune dysregulation, these approaches aim to achieve sustained pathogen clearance, reduce chronic inflammation, prevent tissue remodeling, and ultimately improve lung function and quality of life.

However, several challenges remain. The complexity of polymicrobial biofilms, where different species interact synergistically or antagonistically, necessitates broad-spectrum or targeted therapies that can address multiple pathogens simultaneously (Carolus et al., 2019; Krüger et al., 2019; Vila et al., 2021; Campo-Pérez et al., 2025). The development of antibiotic resistance, even to novel agents, is an ongoing concern, underscoring the need for strategies that minimize resistance development, such as combination therapies or agents that target non-essential bacterial functions like quorum sensing (Wu et al., 2014; Luo et al., 2021; Roque-Borda et al., 2025). Furthermore, the translation of promising *in vitro* and *in vivo* findings into effective human therapies requires rigorous clinical trials, addressing issues of drug delivery, pharmacokinetics, and potential toxicity in the complex pulmonary environment (Cámara et al., 2022; Highmore et al., 2022; Blanco-Cabra et al., 2024). The economic significance of biofilms, with annual global costs in the trillions, highlights the urgent need for effective translational research and frameworks to bridge the gap between academic discoveries and industrial implementation (Cámara et al., 2022; Highmore et al., 2022).

In conclusion, combined anti-biofilm and immunomodulatory therapies represent a critical paradigm shift in managing chronic pulmonary diseases. By addressing both the microbial and host immune components of the disease, these strategies hold immense potential to overcome the limitations of current treatments and offer more effective, long-term solutions for patients suffering from biofilm-mediated immune dysregulation.

## 8 Discussion and future direction

**FIGURE 3:**
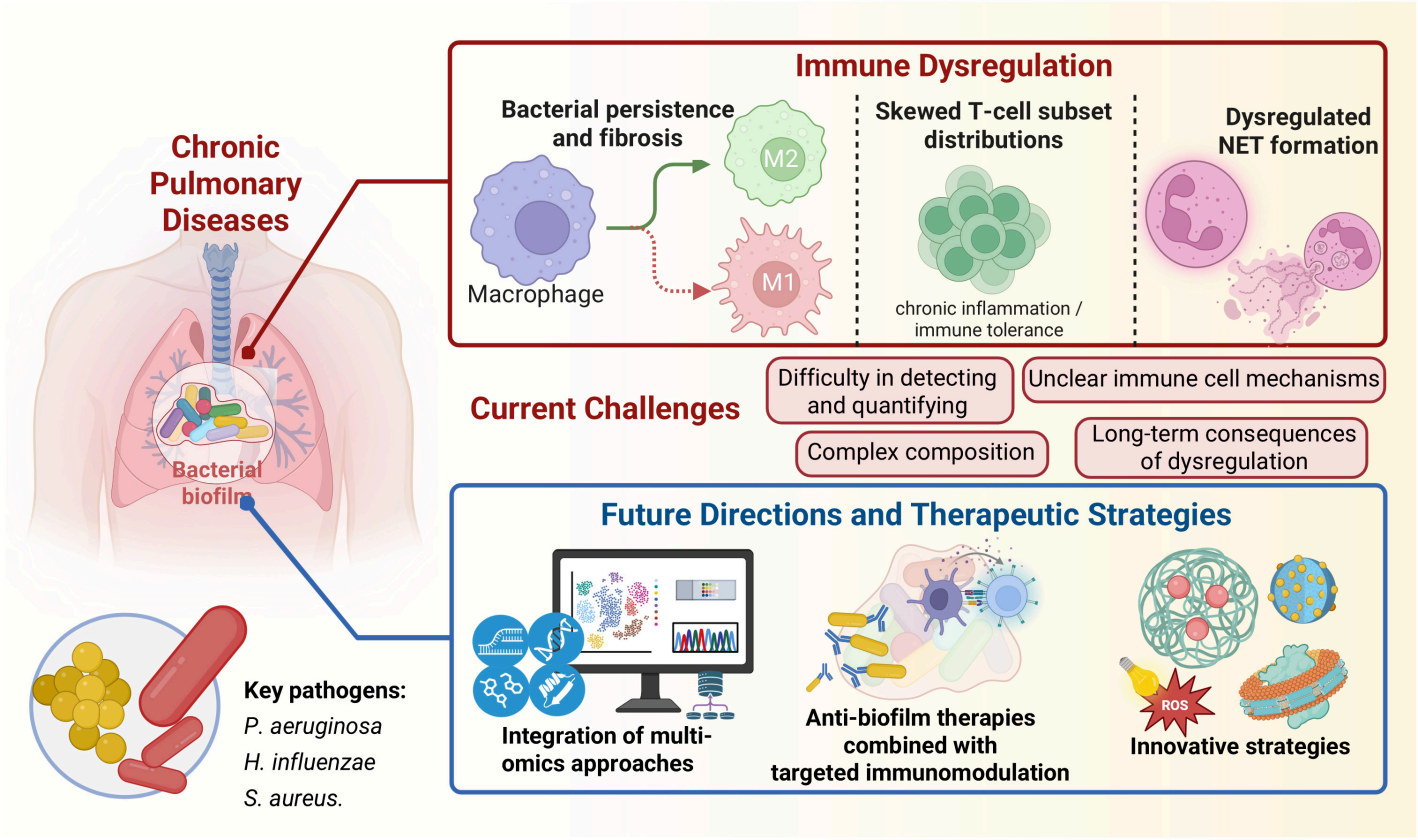
Biofilm-mediated immune dysregulation and therapeutic perspectives in chronic pulmonary diseases.

Microbial biofilms are increasingly recognized as central players in the pathogenesis and progression of chronic pulmonary diseases, mediating profound immune dysregulation that perpetuates inflammation and drives irreversible tissue remodeling. This review has systematically explored the intricate mechanisms by which respiratory biofilms, predominantly formed by pathogens such as *Pseudomonas aeruginosa*, *Haemophilus influenzae*, and *Staphylococcus aureus*, subvert host immunity and contribute to conditions like COPD, bronchiectasis, refractory asthma, and even lung cancer (Figure 3).

The main findings highlight that biofilms actively reshape both innate and adaptive immune responses. In the innate arm, they impair pattern recognition receptor signaling, leading to ineffective pathogen recognition and chronic low-grade inflammation. They dysregulate NET formation, rendering NETs ineffective against biofilm-embedded bacteria while contributing to host tissue damage. Furthermore, biofilms imbalance macrophage polarization, often skewing it toward phenotypes that favor bacterial persistence and fibrosis rather than effective clearance. In the adaptive immune system, biofilms induce skewed T-cell subset distributions, fostering either chronic, ineffective inflammation or immune tolerance, both detrimental to disease resolution. These immune alterations collectively sustain chronic inflammation and drive pathological tissue remodeling, manifesting as emphysema, bronchiectasis, airway hyperresponsiveness, and potentially contributing to oncogenic processes in the lung.

Despite significant advances in understanding biofilm biology and host-pathogen interactions, several research gaps remain. A major limitation is the difficulty in accurately detecting and quantifying biofilm burden *in vivo* in the respiratory tract, as traditional culture methods often fail to capture the biofilm lifestyle. Current methodologies for characterizing biofilm composition, particularly the EPS matrix, are still evolving, and more precise, real-time methods are needed to guide clinical decisions. The complex interplay within polymicrobial biofilms, where different species interact and influence both biofilm dynamics and host immunity, is also not fully understood. Moreover, the precise molecular mechanisms by which biofilms induce specific immune cell polarization states or T-cell differentiation pathways require further elucidation. The long-term consequences of biofilm-mediated immune dysregulation, particularly in the context of lung cancer development, are still emerging and warrant extensive investigation.

Looking ahead, promising future directions emphasize the critical need for multi-omics integration models. By combining genomics, transcriptomics, proteomics, and metabolomics from both microbial and host perspectives, researchers can gain a holistic understanding of the complex interactions at play. Such integrated approaches will be instrumental in identifying novel biomarkers for early detection, disease stratification, and monitoring therapeutic responses. For instance, multi-modal transcriptomic analyses have already revealed metabolic dysregulation and immune responses in COPD, offering potential biomarkers and therapeutic targets (Luo et al., 2024). Furthermore, the development of personalized intervention strategies is paramount. Tailoring anti-biofilm and immunomodulatory therapies based on an individual patient’s specific biofilm composition, immune phenotype, and disease characteristics holds the key to more effective and durable treatments. This could involve precision delivery of antibiofilm agents, such as nanoparticles or enzymes, combined with targeted immunomodulators that restore specific immune functions without causing excessive collateral damage. Advances in nanozyme-driven immune microenvironment remodeling and light-controlled reactive oxygen species generation offer exciting avenues for precise immune regulation and biofilm eradication (Mo et al., 2024; He and Zhang, 2025). Finally, continued investment in sophisticated *in vitro* and *ex vivo* models that accurately mimic the human respiratory environment, coupled with advanced imaging and spectroscopic techniques, will be crucial for accelerating the discovery and validation of new therapeutic targets and strategies. By addressing these challenges, we can move closer to effectively combating biofilm-mediated immune dysregulation and improving outcomes for patients suffering from chronic pulmonary diseases.
